# Life satisfaction and mortality in elderly people: The Kangwha Cohort Study

**DOI:** 10.1186/1471-2458-12-54

**Published:** 2012-01-19

**Authors:** Heejin Kimm, Jae Woong Sull, Bayasgalan Gombojav, Sang-Wook Yi, Heechoul Ohrr

**Affiliations:** 1 Institute for Health Promotion & Department of Epidemiology and Health Promotion, Graduate School of Public Health, Yonsei University, Seoul, Korea; 2 Department of Biomedical Laboratory Science, College of Health Sciences, Eulji University, Seongnam, Korea; 3 Department of Preventive Medicine and Public Health, Kwandong University College of Medicine, Gangneung, Korea; 4 Department of Preventive Medicine, Yonsei University College of Medicine, Seoul, Korea; 5 Department of Biomedical Laboratory Science, College of Health Science, Eulji University, 212, Yangji-dong, Sujoung-gu, Sungnam-Si, Gyeongi-Do 461-713, Korea

**Keywords:** Life satisfaction, Elderly, Mortality, Cardiovascular disease, Quality of life

## Abstract

**Background:**

As well as biomedical risk factors, psychological factors have been reported to be related to mortality rate. The purpose of this study was to examine the relationship between life satisfaction and mortality in elderly people through an 11.8-year follow-up study of a prospective cohort.

**Methods:**

Among 3,600 participants of the Kangwha Cohort Study who survived in 1994, 1,939 respondents of the Life Satisfaction Index (LSI)-A questionnaire were included (men, 821; women, 1118). The mortality risk for the period up to December 2005 was measured using the Cox Proportional Hazard Model.

**Results:**

When the relationship between LSI and mortality was evaluated in men, the unsatisfied group with lower LSI scores showed a significantly higher risk of all-cause mortality (hazard ratio [HR], 1.42; 95% confidence interval [CI], 1.11-1.83) than the satisfied group with higher LSI scores. In women, the unsatisfied group showed a significantly higher risk of all-cause mortality (HR, 1.51; 95% CI, 1.18-1.92) and cardiovascular mortality (HR, 2.23; 95% CI, 1.30-3.85) than the satisfied group.

**Conclusion:**

We found that elderly people with a lower LSI score, regardless of gender, were at risk of increased mortality from all causes, and low LSI score was also associated with cardiovascular mortality.

## Background

Psychological factors and well-being, with no physical risks involved, have been reported to be associated with mortality rates [1-5]. Two Finnish studies found in a 20-year follow-up that people with a low self-reported life satisfaction index (LSI) showed an increased risk of mortality and suicide [4,5]. The relationship between life satisfaction and mortality risk has been examined mostly in Western people; e.g., Finnish elderly people in their 80s and Germans [6,7]. However, a recent Taiwanese study also indicated that life satisfaction is an independent mortality risk factor [8].

In a Finnish prospective study, life satisfaction was associated with a risk of total death in men, but only injury death in women [4]. The Finnish study categorized deaths into total death, disease death, and injury death. Two other socio-epidemiological studies also showed that indicators of impaired quality of life are related to high levels of cardiovascular risk factors and high prevalence of overt cardiovascular disease [9]. In fact, there are few relevant studies that classified causes of deaths by disease [8,9]. In this study, we followed up a prospective cohort of community elderly residents for 11.8 years, and examined the relationship between life satisfaction and risk of mortality from all causes, cardiovascular disease, and cancer in men and women after adjusting for the influences of various known risk factors.

## Methods

### Study population

The primary survey for the Kangwha Cohort was conducted in March 1985 [10,11]. Kangwha County is a rural island in South Korea located 50 km from Seoul with a mostly agricultural population. Its population size was 71,116 in 1993 [11]. The number of Kangwha County residents who were ≥ 55 years of age in February 1985 was 9,378. Among them, 67.9% (or 6,372 residents) participated in the primary survey. In March 1994, the secondary survey, including interviews for demographic characteristics, Mini-Mental State Examination (MMSE), Activities of Daily Living (ADL), and Instrumental Activities of Daily Living (IADL), as well as LSI, was carried out. Body height and weight were measured by trained investigators. Body mass index (BMI) was calculated as the ratio of weight (in kilograms) to height (in meters) squared. This study used data from the secondary survey of the Kangwha Cohort. During the 9-year interval, from March 1985 to March 1994, there were 2,772 deaths, and 3,600 participants survived at the secondary survey in March 1994. Of these, we excluded those who were not followed after the initial survey (n = 13), those who had a stroke or coronary heart disease before the initial survey (n = 23), and those who had no LSI (n = 1219), ADL/IADL (n = 113), or BMI/MMSE (n = 293) data. Thus, the final study population recruited included 1,939 individuals (53.9%, 821 males and 1,118 females). They were followed up for mortality over a maximum of 11.8 years until December 31, 2005. The Institutional Review Board of Human Research of Yonsei University approved the study (approval no. 4-2007-0182).

### Baseline data collection

The investigation team interviewed each subject using a structured questionnaire for demographic characteristics, including age, sex, education, chronic disease conditions, health behaviors (smoking and drinking), MMSE, ADL, IADL, and LSI. Life satisfaction was assessed by the LSI-A index, which was first prepared, validated, and published by Neugarten et al. [12]. The internal consistency reliability coefficient (Cronbach's alpha) was computed at 0.82 in a Korean study with 600 elderly subjects [13]. Of the 20 items in the questionnaire, 12 items were positively worded; 0, 1, and 2 points were assigned to "disagree," "don't know," and "agree" answers, respectively. Eight items were negatively worded with 0 points assigned to the "agree" answer. The scores that could be achieved from LSI-A ranged between 0 and 40 points. Cognitive function was measured using the MMSE [14]. The MMSE test included questions for the following categories: orientation to time, orientation to place, repetition and registration, attention and calculation, recall, naming, repetition, comprehension, reading, writing, and drawing. MMSE scores ranged from 0 to 30. The cut points defined in previous studies were used to categorize the MMSE scores [15,16]. Cognitively impaired persons were defined as those who had a MMSE score below 15.

Disability in ADL was evaluated by the following criteria: bathing or showering, dressing, going to the toilet, transferring (in and out of bed or chair), and eating [17]. For the performance of these activities, participants were asked to answer in one of three categories: perform the activity independently, perform the activity with assistance, and unable to perform the activity. ADL disability was defined as being unable to perform the activity. In other words, if there was an answer of "unable to perform the activity" for any of the seven ADL questions, the respondent was defined as having disability in ADL. In order to define IADL, the following activities were addressed: housework, meal preparation, traveling via car or public transportation, food or clothes shopping (regardless of transport), medication use (preparing and taking correct dose), making telephone calls, and managing money [18]. Each activity was graded on a three-part scale: independent, assistance needed, and dependent. Participants who performed an activity dependently or with assistance were considered as having IADL disability. In other words, if there was an answer of "unable to perform the activity" for any of the 14 IADL questions, the respondent was defined to have disability in IADL. For chronic conditions, study subjects were asked to answer yes or no to the question: "Do you have any chronic disease or past accident or injury for which you feel uncomfortable in your daily lives, including work?"

### Outcome assessment

The outcomes of this research were cause-specific deaths. Data on deaths and their causes from March 1, 1994 to December 31, 2005 were obtained from the Statistics on the Causes of Death in Korea by the Korea National Statistical Office. The International Classification of Diseases, 10th Revision was applied to define the causes of death (I10 to I25 and I60 to I74 for cardiovascular disease, C00-C97.9 for cancer).

### Statistical analysis

LSI scores were grouped into tertiles using the dataset for men and women (N = 1939) to represent 'satisfied' (26-40 points), 'intermediate' (16-25 points), and 'unsatisfied' (0-15 points). Analysis of variance (ANOVA) and chi-square test were used to analyze the statistical differences among the characteristics of the study participants. Because there could be a variation in mortality risk by gender, a stratified analysis was made for men and women.

We used the Cox proportional hazard model to test the relationship between LSI at baseline and subsequent risk of total mortality. Three models were set up to adjust for confounding variables. In Model I, age as a continuous variable was adjusted for. In Model II, in addition to age, the history of chronic disease (ever, never), smoking habits (never, former, 1-19 tobacco/day, and ≥ 20 tobacco/day), alcohol consumption, BMI (as a continuous variable), and education status (no education, elementary, high) were adjusted for. Smoking status and education level were included in the models as dummy variables. In Model III, IADL (normal, abnormal) was further adjusted for. Hazard ratios (HRs) and 95% confidence intervals (CIs) were expressed for the results. A significance level of *p *< 0.05 was used for all tests. Analyses were performed with SAS Windows version 9.1.

## Results

General characteristics of men and women by LSI level are presented in Tables 1 and 2. Mean age was not different between LSI groups in both men and women. In men, the unsatisfied group had significantly more incidences of chronic disease than the satisfied group (*p *< 0.01) (Table 1). The unsatisfied group of men was also significantly less educated (*p *< 0.01), had lower cognitive function, and had more disability in ADL and IADL. In women, although the outcome for IADL was similar to that in men, ADL, cognitive function, and education status were not related with LSI (Table 2). However, smoking, BMI, and chronic disease were significantly associated with LSI in women.

**Table 1:** Baseline characteristics of the study population in the Kangwha Cohort Study in Korean men, according to the LSI

		LSI (N = 821)		F or χ2 value
	
Characteristics	Satisfied	Intermediate	Unsatisfied	
	26-40	16-25	0-15	
	(N = 281)	(N = 316)	(N = 224)	
	Mean ± SD	Mean ± SD	Mean ± SD	
Age (years)	73.2 ± 5.5	72.8 ± 5.3	73.4 ± 5.2	1.01
BMI (kg/m^2^)	21.5 ± 2.8	21.3 ± 3.1	20.9 ± 2.8	3.06*
	N (%)	N (%)	N (%)	
Chronic disease^a^				15.8**
Never	192 (68.3)	205 (64.9)	116 (51.8)	
Ever	89 (31.7)	111 (35.1)	108 (48.2)	
Education				14.9**
No	80 (28.5)	93 (29.4)	95 (42.4)	
Elementary	180 (64.1)	192 (60.8)	115 (51.3)	
High	21 (7.5)	31 (9.8)	14 (6.3)	
Smoking				7.29
Never	82 (29.2)	68 (21.5)	51 (22.8)	
Former	47 (16.7)	58 (18.4)	42 (18.8)	
Current				
1-19 tobacco/day	76 (27.1)	81 (25.6)	63 (28.1)	
≥20 tobacco/day	76 (27.1)	109 (34.5)	68 (30.4)	
Alcohol drinking				4.20
Non-drinkers	132 (47.0)	129 (40.8)	86 (38.4)	
Drinkers	149 (53.0)	187 (59.2)	138 (61.6)	
Cognitive impairment				6.33*
Normal (≥15)	271 (96.4)	295 (93.4)	204 (91.1)	
Impairment (< 15)	10 (3.6)	21 (6.6)	20 (8.9)	
ADL				9.78**
Normal	281 (100.0)	315 (99.7)	219 (97.8)	
Abnormal	0 (0.0)	1 (0.3)	5 (2.2)	
IADL				21.8**
Normal	169 (60.1)	164 (51.9)	88 (39.3)	
Abnormal	112 (39.9)	152 (48.1)	136 (60.7)	

*p < 0.05, **p < 0.01

^a^Study participants were asked to answer yes or no to the question: "Do you have any chronic disease or past accident or injury for which you feel uncomfortable in your daily lives, including work?"

**Table 2:** Baseline characteristics of the study population in the Kangwha Cohort Study in Korean women, according to the LSI

		LSI		F or χ2
		(N = 1118)		value
	
Characteristics	Satisfied	Intermediate	Unsatisfied	
	26-40	16-25	0-15	
	(N = 380)	(N = 376)	(N = 362)	
	Mean ± SD	Mean ± SD	Mean ± SD	
Age (years)	73.5 ± 6.2	73.1 ± 6.0	73.3 ± 5.9	0.42
BMI (kg/m^2^)	22.8 ± 3.6	22.1 ± 3.4	22.3 ± 3.9	3.63*
	N (%)	N (%)	N (%)	
Chronic disease^a^				6.34*
Never	251 (65.0)	227 (60.4)	207 (57.2)	
Ever	129 (34.0)	149 (39.6)	155 (42.8)	
Education				5.60
No	282 (74.2)	292 (77.7)	285 (78.7)	
Elementary	96 (25.3)	80 (21.3)	71 (19.6)	
High	2 (0.5)	4 (1.1)	6 (1.7)	
Smoking				17.6**
Never	334 (87.9)	315 (83.8)	280 (77.4)	
Former	4 (1.1)	8 (2.1)	14 (3.9)	
Current				
1-19 tobacco/day	33 (8.7)	40 (10.6)	56 (15.5)	
≥20 tobacco/day	9 (2.4)	13 (3.5)	12 (3.3)	
Alcohol drinking				0.88
Non-drinkers	348 (91.6)	338 (89.9)	325 (89.8)	
Drinkers	32 (8.4)	38 (10.1)	37 (10.2)	
Cognitive impairment				2.43
Normal (≥15)	286 (75.3)	287 (76.3)	259 (71.6)	
Impairment (< 15)	94 (24.7)	89 (23.7)	103 (28.4)	
ADL				0.70
Normal	377 (99.2)	371 (98.7)	359 (99.2)	
Abnormal	3 (0.8)	5 (1.3)	3 (0.8)	
IADL				9.26**
Normal	157 (41.3)	146 (38.8)	112 (30.9)	
Abnormal	223 (58.7)	230 (61.2)	250 (69.1)	

*p < 0.05, **p < 0.01

^a^Study participants were asked to answer yes or no to the question: "Do you have any chronic disease or past accident or injury for which you feel uncomfortable in your daily lives, including work?"

Table 3 shows the relationship between LSI and mortality in men. After adjusting for age, the unsatisfied group's HR of all-cause mortality, compared to the satisfied group, was 1.61 (95% CI, 1.26-2.04). In Model II, the HR was 1.50 (95% CI, 1.18-1.92). After further adjusting for IADL in Model III, the HR was 1.42 (95% CI, 1.11-1.83). The HR of cardiovascular mortality in Model II was 1.80-fold higher in the unsatisfied group (95% CI, 1.02-3.16) than in the satisfied group. After additionally adjusting for IADL, the HR was still higher, but the result was not significant (HR, 1.63; 95% CI, 0.92-2.89).

**Table 3:** Number of deaths and adjusted hazard ratios of death according to LSI among Korean men

		LSI	
	
Cause of death	Satisfied	Intermediate	Unsatisfied
	26-40	16-25	0-15
	(N = 281)	(N = 316)	(N = 224)
All causes			
No. of deaths	130	186	138
HR (95% CI)^a^	1.00	1.51 (1.20-1.89)	1.61 (1.26-2.04)
HR (95% CI)^b^	1.00	1.49 (1.19-1.87)	1.50 (1.18-1.92)
HR (95% CI)^c^	1.00	1.45 (1.16-1.82)	1.42 (1.11-1.83)
Cardiovascular disease			
No. of deaths	24	28	27
HR (95% CI)^a^	1.00	1.23 (0.71-2.13)	1.74 (1.00-3.01)
HR (95% CI)^b^	1.00	1.18 (0.68-2.04)	1.80 (1.02-3.16)
HR (95% CI)^c^	1.00	1.12 (0.65-1.94)	1.63 (0.92-2.89)
Cancer			
No. of deaths	30	41	26
HR (95% CI)^a^	1.00	1.33 (0.83-2.13)	1.29 (0.76-2.18)
HR (95% CI)^b^	1.00	1.27 (0.79-2.04)	1.19 (0.70-2.05)
HR (95% CI)^c^	1.00	1.29 (0.80-2.08)	1.24 (0.72-2.15)

*Notes*: *HR *hazard ratio, *CI *confidence interval

^a^Adjusted for age (year of recruitment) using the Cox proportional hazard model

^b^Adjusted for age (year of recruitment), history of chronic disease, smoking habits (never, former, 1-19 tobacco/day, and ≥20 tobacco/day), alcohol drinking, body mass index, and education level using the Cox proportional hazard model

^c^Added IADL as covariates in the Cox proportional hazard model

Table 4 shows the relationship between LSI and mortality in women. After additionally adjusting for IADL in Model III, the unsatisfied group had a significantly higher risk of all-cause mortality (HR, 1.51; 95% CI, 1.18-1.92) and cardiovascular mortality (HR, 2.23; 95% CI, 1.30-3.85) than the satisfied group. In both men and women, however, no significant relationship between LSI and cancer mortality risk was observed.

**Table 4:** Number of deaths and adjusted hazard ratios of death according to LSI among Korean women

		LSI	
	
Cause of death	Satisfied	Intermediate	Unsatisfied
	26-40	16-25	0-15
	(N = 380)	(N = 376)	(N = 362)
All causes			
No. of deaths	126	146	155
HR (95% CI)^a^	1.00	1.33 (1.05-1.69)	1.61 (1.27-2.03)
HR (95% CI)^b^	1.00	1.30 (1.02-1.65)	1.53 (1.20-1.95)
HR (95% CI)^c^	1.00	1.30 (1.02-1.66)	1.51 (1.18-1.92)
Cardiovascular disease			
No. of deaths	21	34	38
HR (95% CI)^a^	1.00	1.83 (1.06-3.15)	2.28 (1.34-3.89)
HR (95% CI)^b^	1.00	1.84 (1.07-3.19)	2.28 (1.32-3.91)
HR (95% CI)^c^	1.00	1.84 (1.07-3.18)	2.23 (1.30-3.85)
Cancer			
No. of deaths	13	19	13
HR (95% CI)^a^	1.00	1.53 (0.76-3.10)	1.16 (0.54-2.49)
HR (95% CI)^b^	1.00	1.49 (0.73-3.03)	1.09 (0.50-2.38)
HR (95% CI)^c^	1.00	1.47 (0.72-2.98)	1.06 (0.48-2.31)

*Notes*: *HR *hazard ratio, *CI *confidence interval

^a^Adjusted for age (year of recruitment) using the Cox proportional hazard model

^b^Adjusted for age (year of recruitment), history of chronic disease, smoking habits (never, former, 1-19 tobacco/day, and ≥20 tobacco/day), alcohol drinking, body mass index, and education level using the Cox proportional hazard model

^c^Added IADL as covariates in the Cox proportional hazard model

## Discussion and Conclusions

In this prospective cohort study of Korean elderly subjects, low life satisfaction was associated with long-term mortality. It was also associated with cardiovascular disease-related mortality. These data are largely consistent with the findings of previous studies, such as a Finnish prospective study [4], a Finnish study of elderly people in their 80s [6], a Berlin aging study [7], and a Taiwanese prospective study [8]. The Finnish prospective study showed that low life satisfaction was associated with the increased risk of total death in men, but only of injury death in women [4]. In contrast, the present study shows that the relationship between low life satisfaction and mortality risk was significant in both men and women. Many previous studies of LSI and mortality categorized deaths into total death, disease death, and injury death, whereas the present study evaluated the relationship according to the specific diseases that caused deaths. The present study shows that LSI was associated with a risk of cardiovascular disease, but not of cancer. The outcome suggests a strong relationship between life satisfaction and cardiovascular disease.

The present study used the LSI-A, a 20-item questionnaire, as an assessment tool of quality of life satisfaction. The Taiwanese study also used the LSI-A, and it is known to be useful in assessing overall quality of life satisfaction [8]. Previous studies found that self-reported life satisfaction was associated with cognitive function, formal support, social interaction, social relationships, socioeconomic status, age, marital status, health status, religion, economic status, and/or living arrangements [19-25]. The present study also found that life satisfaction was associated with chronic disease conditions, education level, cognitive function, and disability in ADL and IADL in Korean elderly men.

This study has several limitations. First, we adjusted BMI as a covariate. However, other classical biological risk factors for cardiovascular-related death, such as hypertension, diabetes, and dyslipidemia, were not included in this study. Second, non-inclusion of variables, such as depressive symptoms, sense of well-being, and social support, may be a limitation, although well-being is a dimension measured by the LSI-A in a comprehensive sense. Third, deaths from diseases other than cardiovascular disease, cancer, and all causes could not be analyzed due to an insufficient number of cases. More causes of death need to be analyzed in further studies. Fourth, selection bias is often an issue in studies of life satisfaction. The subjects in this study were an elderly group residing in the same administrative and geographical community, and approximately 85% of them engaged in agriculture, thus there was a great social and cultural similarity among them.

In conclusion, our findings indicate an association between low life satisfaction and long-term mortality among elderly people, in particular cardiovascular disease mortality. Further studies are necessary to describe the relationship between life satisfaction and mortality risk for a wider range of diseases.

## Abbreviations

ADL: Activities of Daily Living; BMI: Body mass index; CI: Confidence interval; HR: Hazard ratio; IADL: Instrumental Activities of Daily Living; LSI: Life Satisfaction Index; MMSE: Mini-mental state examination.

## Competing interests

The authors declare that they have no competing interests.

## Authors' contributions

All authors participated in drafting of the manuscript. HK performed the analysis and interpretation of data. JWS participated in planning the design and interpretation of data. BG and SWY participated in data analyses and interpretation. HO conceived the idea of the study, planned the design, collected data, and participated in interpretation of the data. All authors read and approved the final manuscript.

## Pre-publication history

The pre-publication history for this paper can be accessed here:

http://www.biomedcentral.com/1471-2458/12/54/prepub
